# An Automatic Approach Designed for Inference of the Underlying Cause-of-Death of Citizens

**DOI:** 10.3390/ijerph18052414

**Published:** 2021-03-02

**Authors:** Hui Ge, Keyan Gao, Shaoqiong Li, Wei Wang, Qiang Chen, Xialv Lin, Ziyi Huan, Xuemei Su, Xu Yang

**Affiliations:** 1 Chinese Center for Disease Control and Prevention, Beijing 102206, China; gehui@chinacdc.cn (H.G.); lisq@chinacdc.cn (S.L.); wangwei2@chinacdc.cn (W.W.); chenqiang@chinacdc.cn (Q.C.); 2 School of Computer Science and Technology, Beijing Institute of Technology, Beijing 100081, China; 3220180795@bit.edu.cn (K.G.); 3220201066@bit.edu.cn (X.L.); 3220200891@bit.edu.cn (Z.H.)

**Keywords:** cause-of-death inference, automatical, public heath, medical service

## Abstract

It is very important to have a comprehensive understanding of the health status of a country’s population, which helps to develop corresponding public health policies. Correct inference of the underlying cause-of-death for citizens is essential to achieve a comprehensive understanding of the health status of a country’s population. Traditionally, this relies mainly on manual methods based on medical staff’s experiences, which require a lot of resources and is not very efficient. In this work, we present our efforts to construct an automatic method to perform inferences of the underlying causes-of-death for citizens. A sink algorithm is introduced, which could perform automatic inference of the underlying cause-of-death for citizens. The results show that our sink algorithm could generate a reasonable output and outperforms other stat-of-the-art algorithms. We believe it would be very useful to greatly enhance the efficiency of correct inferences of the underlying causes-of-death for citizens.

## 1. Introduction

It is very important to have a comprehensive understanding of the health status of a country’s population, which will help to develop corresponding public health policies. Correct inference of the underlying cause-of-death for citizens is essential to achieve a comprehensive understanding of the health status of a country’s population. Thus, inference of the underlying cause-of-death of citizens is of great significance.

On one hand, a correct understanding of the overall composition of the social average population mortality rate and the underlying cause-of-death of citizens is not only helpful to make up for the shortcomings of medical and health services, to improve the abilities of medical treatment, and to improve comprehensive modern medical systems but also involves the study and formulation of social pension policies [1], which has far-reaching significances for maintaining long-term social stability and for pursuing the happiness of national life.

On the other hand, inference of the underlying cause-of-death of citizens also contributes to the prevention and treatment of infectious diseases and to response to major health emergencies.

Since ancient times, inference of the underlying cause-of-death is a technology that integrates empiricism and rationalism. On the one hand, in the process of criminal case investigation, accurate inference of the underlying cause-of-death of the dead not only is an inevitable requirement to find out the truth of the case and to arrest the murderer but also helps to determine the criterion of sentencing. On the other hand, from ancient times to the present, accurate inference of the underlying cause-of-death of patients is not only a final judgment in the medical process but also a teaching experience for the development of medical skills.

However, traditionally, determination of the underlying cause-of-death of citizens is mainly based on experience of medical staff. The disadvantages are obvious [2,3,4,5,6,7]:

1. The accuracy of the results depends heavily on the experience of the medical staff;
2. The subjective judgment of the same medical personnel will be affected by the objective environment, resulting in a certain fluctuation in the accuracy of the judgment;
3. Due to the lack of automation, a lot of manpower and time need to be invested. Thus, it is not an effective way.

Many scholars begin to advocate that we should pay attention to inference of the underlying cause-of-death, to establish a new inference model, to improve the efficiency of the inference of the cause-of-death, and to provide a better data basis for the study of the underlying cause-of-death [8,9].

In this work, we aim to construct an automatic way to determine the underlying cause-of-death of citizens.

The following is organized as follows: Section 2 will discuss the literatures; Section 3 gives the problem definition; the sink algorithm is described in Section 4; the results are shown and discussed in Section 5; and a conclusion is given in Section 6.

## 2. Related Works

As we have mentioned earlier, correct inference of the underlying cause-of-death for citizens is of great importance for understanding the public health status of a country and many researchers have been dedicated to this topic.

In 1991, Shaw-Hwa Lo proposed to construct an incomplete “cause-of-death chain” during clinical treatment according to the patient’s condition and to build a prediction algorithm to predict the probability of a patient’s cure and survival [10]. Also in 1991, Sadovnick et al. presented their study, which found that the number of cases that did cause death directly due to multiple sclerosis accounted for less than 47.1% of the total sample and that there were a large number of multiple sclerosis cases that eventually died for other reasons [11] by studying the data of multiple sclerosis patients recorded in some clinics in Britain between 1972 and 1988. In 1996, Garne et al. [2] presented their work, which analyzed official reports of deaths from breast cancer in Malmo, Sweden, from 1964 to 1992 and found that at least 4.6 percent of death cases’ real causes-of-death were different from the officially reported cause-of-death, with the proportion of inconsistent cases increasing as the age of death increased. In 1998, according to Sathiakumar et al.’s research, even the same cause-of-death chain, in the case of multiple medical personnel alone judgment, often produces different inference results [12]. Also in 1998, Donald M.LloydJones et al., by organizing medical workers to proofread the underlying causes-of-death reported by death certificates, found that at least 12.15% of cases over the age of 85 had been wrongly identified as coronary heart disease [13]. There are other researches that also indicate the existence of the same phenomena.

This not only suggests that traditional methods that perform inference based on medical staff’s experiences might not be a sufficiently efficient method but also implies that the outcome of the final death of clinical cases is rarely caused by one cause and that the potential role of other factors cannot be ignored.

Therefore, many researchers began to investigate the influence of other factors on the correct inference of underlying causes-of-death, such as the physical health characteristics of the case, including age and sex, and the geographical characteristics [4,5,6,7,14,15].

In 2018, Alexandre Boumezoued et al., in order to study the influence of population mortality and cause-of-death on social development trend, constructed a statistical model by using the theory of deterministic and random population dynamics model [1]. They used mathematical statistics to study a variety of diseases and gave some inspiration to a later study of cause-of-death inference considering the influence of various factors. In 2019, Piotr Sliwka proposed a non-Gaussian scalar filter model to determine life expectancy based on different cause-of-death ratios for cancer and cardiovascular disease [16]. They found that, even in the same type of disease, life expectancy damage to patients of different genders varies. In the same year, by using the relevant data of a certain hospital, Xiaoping You [17] found that there were some differences in the distribution of the underlying causes-of-death among residents of the same region with respect to different ages and different genders.

Besides taking more factors into consideration when performing inference of the underlying cause-of-death, researchers also tried to introduce more innovative methods in this domain.

In 2013, Samuel Danso et al. proposed to formalize the text data on the death report and to realize an automatic method obtaining multiple causes-of-death by using automatic text classification technology [18]. In 2019, Louis Falissard et al. designed a software based on deep learning technology to automatically transform the text data of cause-of-death into standard patient-type coding from the data contained in the death report [19]. On this basis, they studied the inference model based on deep artificial neural network, which obtained 75% accuracy. However, similar to Samuel Danso et al.’s research, because the inference model of a deep artificial neural network needs a lot of training data to achieve certain accuracy, the model proposed by Louis Falissard et al. relies on manual processing of a large number of preset data.

We summarize all those researches in Table 1. According to the analysis of the literature, we could concluded the following:

1. The basic characteristics of citizens, such as gender, age, region, and history of related diseases, are of great significance to the judgment of the underlying cause-of-death;
2. In order to remove the influence of human subjective factors in the process of underlying cause-of-death inference, it is necessary to construct an automatic method.

## 3. Problem Definition

This work focuses on constructing an automatic flow to determine the underlying causes-of-death of citizens based on analysis of the cause-of-death chain. After the death of a citizen, according to the development of the citizen’s disease, straightening out the order and recording it, it is the cause-of-death chain of that citizen.

**Figure 1:**
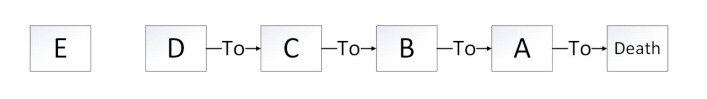
Illustration of the cause-of-death chain.

As shown in Figure 1, the cause-of-death chain usually contains 4 items: A,B,C, and *D*, which might be different diseases, physical injuries, or complications, coded according to WHO’s ICD-10 [20]. In the cause-of-death chain, *A* is the direct cause-of-death. In addition, sometimes, there are other things that have important implications for a citizen’s death but are not recoded in the cause-of-death chain, as the past medical history, which we represent as *E* in Figure 1.

The problem is to determine which one is the underlying cause-of-death among A,B,C,D, and E. The underlying cause-of-death could be divided into two kinds: internal and external causes. If the underlying cause-of-death is of external cause, then the underlying cause-of-death might be the same one as the direct cause-of-death, that is, *A* [21,22].

In the majority of cases, the underlying cause-of-death is among A,B,C, and D, but in some occasional cases, it could also be from *E*.

## 4. Sink Algorithm for Main Cause-Of-Death Inference

In this section, we present the sink algorithm designed for underlying cause-of-death inference motivated by mathematical statistics.

### 4.1. Basic Concept

According to the realization presented in Section 2, we defined several concepts.

Regional Death Cause Proportion (RDCP): It is defined as

(1) $$rdcp_{ij}=\frac{n_{ij}}{N_{j}}$$

where $rdcp_{ij}$ is the regional death cause proportion for cause *i* in region *j* in a certain time span, while $n_{ij}$ is the number of cases where cause *i* is the underlying cause-of-death in region *j* in that time span and $N_{j}$ is the total number of death in region *j* in the time span.

Regional death cause proportion represents the probability that a cause would be the underlying cause-of-death in a region.

Conditional Death Cause Proportion (CDCP): It is defined as the conditional probability that cause *i* is the underlying cause-of-death of a citizen under the condition that cause *i* appeals to that citizen’s cause-of-death chain.

### 4.2. Data Exploration

**Figure 2:**
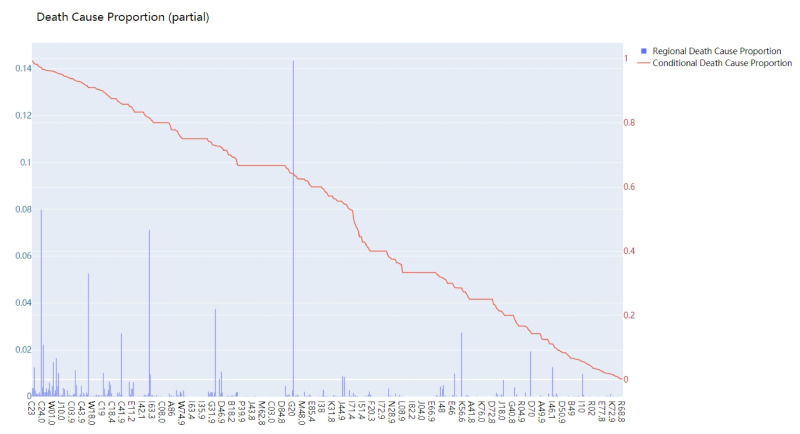
Statistical result of the death cases in City X.

Figure 2 shows the statistical result of RDCP and CDCP of City X located in northern China (only partially). The horizontal coordinate represents different causes-of-death coded in ICD-10. The vertical coordinate represents the value of RDCP and CDCP for different causes-of-death.

It could be seen in Figure 2 that

1. RDCP, to some extent, reflects the health hazards of various diseases in a certain region. Some causes that have larger RDCPs have larger probabilities to be the underlying cause-of-death for citizens in that region. For example, in Figure 2, the RDCP values for I25.1 (14.36%), C34.9(7.99%), and I69.3 (7.12%) are the most significant ones, so they might be more likely to be the underlying cause-of-death for citizens in that region;
2. CDCP represents the possibility that a disease becomes the underlying cause-of-death for a citizen. According to our analysis, some of the causes have CDCPs almost equal to 1, which means that, if a citizen unfortunately suffers from the disease, it is almost certain that the underlying cause-of-death of the citizen is that disease.

We could comment that, due to regional causes, some specific causes are much more likely to cause death than other types of causes in that certain region. At the same time, t some causes have very high CDCP, meaning that, for individual citizens, once they catch that cause, it might be the underlying cause-of-death for those citizens.

**Figure 3:**
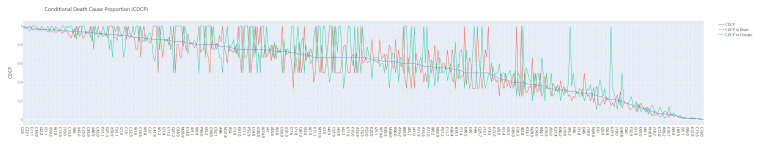
Illustration of the relationship between gender and Conditional Death Cause Proportion (CDCP) in City X.

Figure 3 shows the CDCP for causes with respect to different genders. The blue line shows the CDCP for the whole population, while the green line shows the CDCP for females and the red line shows the CDCP for males. We could see that some causes, for example, J60, has a very high CDCP for males (81.69%), while the CDCP for females is very low, as we know that it heavily correlated to the gender distribution in the related occupation. In addition, some causes have much larger CDCPs for females compared to males. We could concluded that some of the causes have very strong correlation with gender factors.

**Figure 4:**
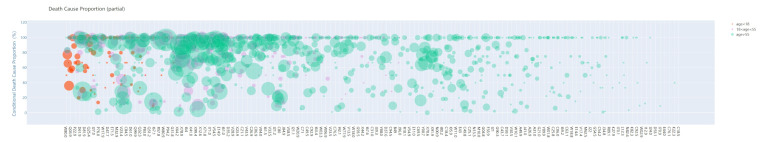
Illustration of the relationship between age and CDCP in City X.

Figure 4 shows the CDCP for causes with respect to different age groups. We divide them into three groups: [age≤18], [18<age<55], and [age≥55]. The group [age≤18] is represented by a red bubble; the purple bubble represents group [18<age<55]; and a green bubble represents group [age≥55]. The radius of each bubble represents the total number of people in that age group suffering from this cause.

It could be concluded from Figure 4 that

1. Different causes have different susceptible populations;
2. CDCP for a cause is varied for different age groups;
3. CDCP is not positively correlated with age;
4. The larger the bubble radius, the more universal the CDCP of a cause. Because the larger the bubble radius corresponding to a cause, the more samples of that cause is in this age group, the more persuasive it is.

### 4.3. The Sink Algorithm

According to our investigation of the literatures and the exploration of data, we designed a sink algorithm for inference of underlying cause-of-death for citizens.

The idea behind the sink algorithm is that we treat a citizen as a boat on the river:

- Initially, the citizen is a healthy person, just as the boat is in pristine status and floats on the river normally;
- As time goes on, different causes appear on the cause-of-death chain of a citizen, as one by one, stones are placed in the boat. Each stone corresponds to a cause in the cause-of-death chain;
- At last, the citizen dies, corresponding to the boat sinking as a result of all the stones in it.

There are several things we need to clarify:

- The boat sinks as a result of all the stones put in it, neither because of the first stone only nor because of the last stone only;
- We could treat the main cause of the boat’s sinking as the heaviest stone put in the boat as the underlying cause-of-death of a citizen;
- Different boats have different original characteristics, which would have influences on the sinking boat. The same goes for the citizens, as they have different statuses;
- Besides the original characteristics and the stones, there are other factors that affect it’s sinking, say environment, which could correspond to the past medical history of a citizen.

Thus, we could formulize the death process of a citizen as follows:

(2) R=A+B+C+D+E−ϵ(α+β+…)

where *R* represents a citizen dying (or a boat sinking); A,B,C, and D are different causes recorded in the cause-of-death chain (different stones); *E* is the past medical history; and α,β and other parameters are used to describe the various statuses of a citizen.

The underlying cause-of-death would be defined as the heaviest one among A,B,C,D, and *E*. In this work, we define the CDCP of a cause as the weight.

As a matter of fact, the A,B,C, and D four causes in the cause-of-death chain often constitute a potential causal chain. The A,B,C,D, and *E* five causes generally have some implicit pathological connection. Over the course of time, the causes in the front of the cause-of-death chain are likely to be the cause of the subsequent causes. The CDCP of those causes are likely to be different. Thus, it makes sense to pick the cause with the largest CDCP as the underlying cause-of-death.

To control the value range of the output variables of the CDCP function, to suppress the phenomenon that the results of different causes are too different, and to suppress the adverse interference of a few cases to the inference process, the CDCP formula we finally adopt in this work is as follows:

(3) $$CDCP_{i}=\log_{2}\frac{death\_count[i][age][gender]}{occur\_count[i][age][gender]}*10$$

where death_count[i][age][gender] represents the number of samples, where cause *i* is the underlying cause-of-death of that age group and gender group, while occur_count[i][age][gender] represents the number of samples, where cause *i* appears on a dead citizen’s cause-of-death of that age group and gender group.

In this work, we divided the samples into three ages groups: [age≤18], [18<age<55], and [age≥55]. There are two gender groups: male and female.

## 5. Experiments and Results

The program is designed with Python 3.5. The database is MySQL 8.0. The dataset includes almost 50,000 records from City X in northern China.

We use the cross-validation method to verify the effect of our sink algorithm. The whole dataset is split into 17 subsets. Each turn, 16 subsets are used to train the algorithm while 1 subset is used as the test set. After 16 turns, the average results are used as the final result.

In order to use the sink algorithm, we first need to calculate the CDCP value for different causes.

Table 2 shows the result of CDCP for several causes coded in ICD-10. It could be seen that age and gender have obvious influences on the result.

**Figure 5:**
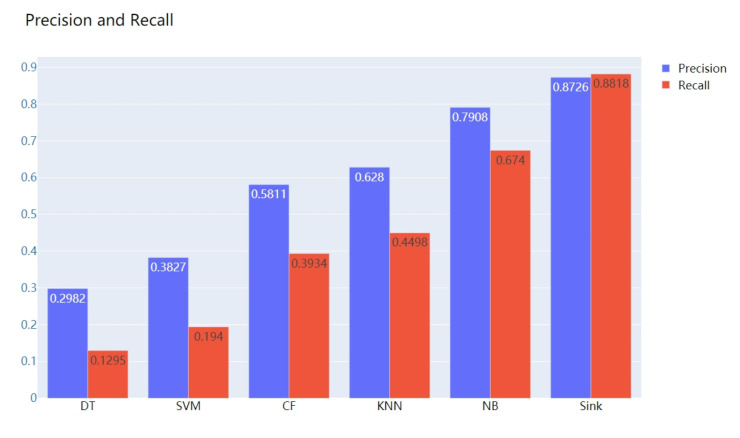
Comparison with state-of-the-art methods.

In order to testify the result of our sink algorithm, we have chosen several other state-of-the-art methods used for classification, such as SVM (Support Vector Machine), DT (Decision Tree), CF (Collaborate Filter), KNN (K Nearest Neighbor), and NB (Naive Beyes). The comparison result is shown in Figure 5.

As shown in the figure, sink algorithm could generate the highest precision and recall, which verifies that our method could achieve the best performance in the inference of underlying cause-of-death for citizens.

**Figure 6:**
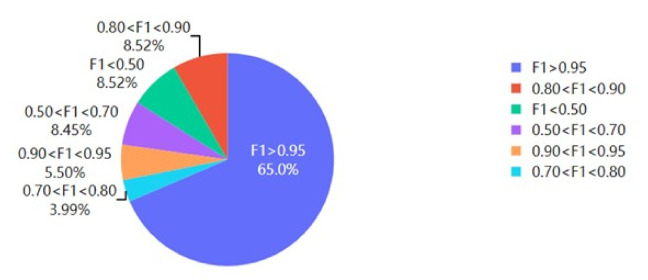
F1-score for different ICD-10 causes.

In order to evaluate the general applicability of our method for different causes, we calculated the accuracy and recall of our sink method on different ICD-10 causes and used F1-score as the final metric. The result is shown in Figure 6.

It could be seen that, for 65% of causes, the F1-score is larger than 0.95. The proportion that has an F1-score less than 0.7 is only 16.97%. Thus, this proves that our sink method has a high general applicability.

## 6. Discussion

In this work, we presented our effort at constructing an automatic way to implement accurate inference of underlying cause-of-death for citizens. We introduced a sink algorithm to achieve that goal. In order to implement an automatic way to perform inference for the underlying cause-of-death, we investigated the cause-of-death chain problem and decided to formulized it as a classification problem. Therefore, we chose typical classification algorithms such as SVM (Support Vector Machine), DT (Decision Tree), CF (Collaborate Filter), KNN (K Nearest Neighbor), and NB (Naive Beyes) for comparison. The results show that our algorithm could generate better results when compared to some state-of-the-art algorithms.

The idea of SVM is to distinguish different cause-of-death categories to the greatest extent, to maximize the data interval of different types of underlying cause-of-death in the training set, and finally to transform it into a convex quadratic programming problem. However, the number of causes-of-death is very large while the dataset of the cause-of-death chain is limited in this case: only according to a certain number of training datasets to calculate the relationship between the causes-of-death and the underlying cause-of-death; it is almost impossible to obtain reliable results. The DT method takes the characteristics of age, gender, and cause-of-death as input and takes one of them as the root node to predict the underlying cause-of-death. However, it is also not efficient due to the limited dataset. KNN algorithm uses age, gender, and cause-of-death as axes to construct a high dimensional space, and individual cases are data points in high dimensional space. To predict the underlying cause-of-death, the k data points closest to the individual case that to be inferred are selected. On the one hand, the inference results depend to some extent on the amount of similar data. On the other hand, it is difficult to design the influence of various factors carefully and to consider the pathological principle. The NB method takes the cause-of-death as a combination of discrete variables. It is assumed that all causes in the cause-of-death chain are independent variables, and different underlying cause-of-death are used as classification categories. The probability of occurrence of different categories and the conditional probability of occurrence under the current cause-of-death are obtained. When inferring individual cases, the classification with large probability value is used as the inference result. Because of the complexity of the variety of causes, it is difficult to ensure uniform distribution of different types of data in the process of counting the probability of each cause’s appearance, and at the same time, there is a high requirement for the size of the training set. The idea of CF is to “find similarities and connections ”. If the cause-of-death chain in one case is exactly the same or very similar to that in another case, the underlying cause-of-death in both cases is most likely the same. Therefore, by constructing various variables for the cause-of-death as different dimensions, we can use CF analysis to find the closest case data and then infer the most likely underlying cause-of-death. However, the data in the cause-of-death chain are very limited, which is different from the conventional massive data environment in the recommendation system. The cause category itself has the characteristics of complex classification, resulting in the CF method being not applicable.

## 7. Conclusions

The sink algorithm presented in this paper is built based on probability calculation. We considered the influence of other factors in the inference of an underlying cause-of-death. Although our method has a better result when compared to other state-of-the-art methods, it also has some limitation. In some special cases, such as when there is no intuitive pathological causality between the cause-of-death in the cause-of-death chain, the performance of our sink algorithm is limited. We can make improvements to handle those limitations in our future work.

Inference of underlying causes-of-death for citizens plays a very important role in the public health and medical service domain. However, the inference of the underlying cause-of-death has not been automated in an efficient way yet. Compared with the commonly used method where rules and conditions are concerned to make an inference of underlying causes-of-death, our sink algorithm provides a way to do it automatically and more efficiently and could be a reference and aid for current medical practice.

## Figures and Tables

**Table 1:** Summary of the presented related researches.

Research	Publication Year	Main Findings
S. Lo [10]	1991	Proposed to construct incomplete “cause-of-death chain”
		during clinical treatment
Sadovnick et al. [11]	1991	Found that there were a large number of multiple sclerosis cases
		that eventually died for other reasons
Garne et al. [2]	1996	Found there were difference between the officially reported
		cause-of-death and the real one
Sathiakumar et al. [12]	1998	Different medical staff will produce different inference
		results of the same cause-of-death chain
D. Lloydjones et al. [13]	1998	Found proof that there is misjudging of the
		cause-of-death through manual methods
S. Danso et al. [18]	2013	Proposed to use automatic text classification technology
A. Boumezoued et al. [1]	2018	Constructed a statistical model, and gave some
		inspiration on considering the influence of various factors
P. Sliwka [16]	2019	Proposed a non-Gaussian scalar filter model, and
		indicated that gender factor matters
X. You [17]	2019	Presented that age and gender factors’ influence
L. Falissard et al. [19]	2019	Presented a method based on deep learning technology

**Table 2:** Illustration of the CDCP values for several causes.

ICD-10	CDCP	Male	Female	age≤18	18<age<55	age≥55
J18.9	0.28514949	0.2663358	0.31307550	0.303030	0.2550335	0.2861578
I61.9	0.8600605	0.8740831	0.83730158	0.285714	0.88851351	0.85574092
J98.4	0.14804722	0.1426710	0.1548254	0.1176470	0.122362	0.14930489
A41.9	0.01851851	0.016194331	0.02162162	0.1379310	0.0133333	0.01447368
